# A comparative analysis of rapid diagnostic tests and enzyme-linked immunosorbent assays for dengue diagnosis: a retrospective study in a tertiary care hospital in India

**DOI:** 10.1128/spectrum.00073-26

**Published:** 2026-10-06

**Authors:** Akash Randive, Shripad Taklikar, Anita Ramchandran

**Affiliations:** 1 Lokmanya Tilak Municipal Medical College, and General Hospital 29649 , Mumbai, India; 2 Department of Microbiology, Lokmanya Tilak Municipal Medical College, and General Hospital 29649 , Mumbai, India; UCI Health, Orange, California, USA

**Keywords:** dengue diagnosis, NS1 antigen, rapid diagnostic test (RDT), enzyme-linked immunosorbent assay (ELISA), diagnostic accuracy, seasonal variation

## Abstract

Dengue fever remains a major public health concern in India, where rapid and accurate diagnosis is essential for effective patient care and outbreak control. While enzyme-linked immunosorbent assay (ELISA) serves as the diagnostic standard, NS1 antigen-based rapid diagnostic tests (RDTs) offer faster and more accessible alternatives, particularly in resource-limited settings. This retrospective study analyzed 3,488 patient samples tested using both NS1Ag-RDT and NS1Ag-ELISA between January 2023 and December 2024. Using ELISA as the reference standard, the diagnostic accuracy of NS1Ag-based RDT was determined. 1,036 cases were ELISA-positive and 2,452 cases were ELISA-negative; the RDT identified 988 true positives, 2,274 true negatives, 178 false positives, and 48 false negatives. The NS1Ag-RDT demonstrated a sensitivity of 95.36% and specificity of 92.74%, indicating strong agreement with NS1Ag-ELISA. These findings support the utility of NS1Ag-based RDTs as reliable tools for early dengue diagnosis, while highlighting the need for multicentric studies incorporating RT-PCR to further validate diagnostic performance across diverse settings.

**IMPORTANCE:**

Dengue virus infection continues to represent a public health burden in endemic regions, where timely and accurate diagnosis is crucial for effective patient management and outbreak control. In this context, NS1 antigen-based diagnostic assays play an essential role in routine clinical practice, particularly in resource-limited settings due to limited access to molecular diagnostics. This study provides robust, real-world evidence on the diagnostic performance of NS1 antigen-based rapid diagnostic tests through a large retrospective analysis of 3,488 patient samples collected over a 2-year period at a tertiary care center in India. The findings demonstrate high sensitivity and specificity of NS1Ag-RDTs when compared with NS1Ag-based enzyme-linked immunosorbent assay (ELISA), and further highlight seasonal variations in diagnostic accuracy, offering clinically relevant insights that can inform diagnostic strategies during peak transmission periods. This work strengthens the evidence base supporting NS1-based diagnostics in dengue-endemic settings and contributes data of significant relevance to virology-focused research and public health practice.

## INTRODUCTION

Dengue is a mosquito-borne viral infection caused by four serotypes (DENV-1 to DENV-4), with clinical presentations ranging from mild febrile illness to life-threatening complications such as dengue hemorrhagic fever (DHF) and dengue shock syndrome (DSS). Dengue threatens over 2.5 billion people globally, with an estimated 390 million infections annually, of which only 92 million are symptomatic (1, 2).

India remains hyperendemic, reporting a total of 233,519 cases and 297 deaths in 2024 alone, and the state of Maharashtra itself recorded 19,385 cases and 40 deaths in the same year. This indicates a rising trend in the number of cases and deaths due to dengue since 2019, as indicated in the data published by the National Vector Borne Disease Control Programme of India (3).

Given the overlap of clinical features with other febrile illnesses such as malaria, chikungunya, and typhoid (4, 5), laboratory confirmation plays a pivotal role in timely diagnosis and management. WHO and NVBDCP guidelines recommend NS1-antigen detection during the acute phase (first 5 days), followed by IgM/IgG serology. The two most accessible diagnostic modalities are enzyme-linked immunosorbent assay (ELISA) and rapid diagnostic tests (RDTs). ELISA is considered more reliable under controlled conditions but is resource-intensive. RDTs, while faster and more deployable in peripheral settings, have shown variable diagnostic accuracy (4–7).

This study aims to evaluate the diagnostic accuracy of NS1Ag-RDT in comparison to NS1Ag-based ELISA, where ELISA is taken as the reference standard for confirming diagnosis of dengue cases. Previous studies comparing NS1- and IgM-based RDTs and ELISAs have reported varying sensitivity and specificities. The findings from this study may offer evidence-based guidance for diagnostic decision-making and resource allocation within India’s public health framework.

The aims and objectives of this study are to perform a comparative analysis of dengue rapid diagnostic tests (RDTs) and enzyme-linked immunosorbent assay (ELISA) by evaluating the diagnostic accuracy (sensitivity and specificity) of NS1 antigen-based RDT against NS1 antigen-based ELISA as the reference standard, and to assess seasonal variation in the diagnostic performance of NS1 antigen-based RDT compared to NS1 antigen-based ELISA, analyzing fluctuations in sensitivity and specificity across summer, monsoon, autumn/post-monsoon, and winter months.

## MATERIALS AND METHODS

A retrospective cross-sectional study was designed comparing the diagnostic performance of rapid diagnostic tests (RDTs) and enzyme-linked immunosorbent assays (ELISA) in the diagnosis of dengue. The primary source of data were the diagnostic registers maintained by the Department of Microbiology at a tertiary care hospital, Lokmanya Tilak Municipal Medical College and General Hospital, Mumbai, covering the period from January 2023 to December 2024. The registers document weekly and monthly test records of all patients screened for dengue using both RDT and ELISA. Data are presented in a 2 × 2 tabular contingency framework, including the result of RDT (positive/negative), result of ELISA (positive/negative), final diagnostic classification, and date of testing.

This pre-structured data allowed for direct extraction and analysis without the need for constructing the diagnostic matrix *de novo*. Data extraction was carried out manually from the physical registers without recording any personal identifiers. Extracted entries were anonymized and stored in a secure, password-protected spreadsheet accessible only to the research team.

### Study population

All patients who had been diagnosed with dengue within the first 5 days of symptom appearance by the Department of Microbiology at the Lokmanya Tilak Municipal General Hospital were included in the study. Individuals were included if their clinical features were consistent with a suspected dengue case (8) and if they had undergone both NS1Ag-RDT and NS1Ag-based ELISA testing during the acute febrile phase. Any patients who had not undergone both aforementioned tests were excluded from the study, thus ensuring a uniform dataset and allowing an accurate comparative evaluation of the two diagnostic tests.

For this study, NS1Ag-based ELISA-positive patients were considered as confirmed dengue cases, in accordance with the NVBDCP, Government of India Initiatives for Dengue and Chikungunya, which states: “As soon as a dengue case is confirmed by serological test (NS1 or IgM Capture ELISA Test), the district vector borne Disease Control Officer/District Chief Medical Officer should take appropriate action” (9).

### Specimen collection and processing

Venous blood samples were collected from all patients presenting with suspected dengue fever at the time of clinical evaluation. Serum was separated from whole blood on the day of collection by centrifugation. All specimens were tested fresh without prior storage or refrigeration. Both the rapid diagnostic test and the ELISA were performed on the same serum specimen on the same day as collection, ensuring comparability of results from a single sample.

### Rapid diagnostic test for dengue NS1 antigen detection

#### Kit details

Dengue NS1 antigen was detected in serum using the Rapicheck Dengue Antigen NS-1 Test (Rapid Diagnostic Test), Manufacturer name: Oscar (Catalog no. RP07-0823-001, Expiry date: 07/2025), a lateral flow immunochromatographic assay for the qualitative detection of dengue virus NS1 antigen in human serum. The same kit brand and generation were used consistently throughout the entire study period (January 2023 to December 2024) without change.

#### Procedure

All test devices and serum specimens were allowed to reach room temperature (15–30°C) prior to testing. The test device was removed from its sealed foil pouch and placed on a clean, flat, horizontal surface. Serum was applied to the designated sample well of the device using the dropper provided in the kit. Assay buffer was then added to the sample well in the volume specified by the kit insert. The device was left undisturbed at room temperature and results were recorded at 15–20 min from the time of specimen and buffer addition. Results were not read beyond the 20-min window.

#### Interpretation of results

A positive result was defined by the appearance of two distinct colored bands: one at the Control region (C) and one at the Test region (T), irrespective of the intensity of the test band. A negative result was defined by the presence of a single colored band at the Control region (C) alone, with no visible band at the Test region (T). Any result in which no band appeared at the Control region (C) was classified as invalid, and the test was repeated using a fresh device and a fresh aliquot of the same specimen.

### Enzyme-linked immunosorbent assay for dengue NS1 antigen detection

#### Kit details

Confirmatory testing was performed using the Qualisa Dengue NS-1 ELISA (Catalog no. 161-065, Expiry Date: 06/25), manufacturer name: OSCAR, a sandwich enzyme-linked immunosorbent assay for the qualitative detection of dengue NS1 antigen in human serum or plasma. The assay is configured on a 96-well microplate format with pre-coated wells. The same kit brand and generation were used consistently throughout the entire study period (January 2023 to December 2024) without change.

#### Procedure

All kit reagents were brought to room temperature and prepared according to the manufacturer’s instructions prior to the start of each assay run. Pre-coated 96-well microplate strips were used for all runs. Serum specimens and the kit-provided positive and negative controls were pipetted into their respective wells in the volumes specified in the kit insert. The plate was sealed and incubated at 37°C for the duration stipulated by the manufacturer. Following incubation, the wells were aspirated and washed with the wash buffer provided to remove unbound material. The enzyme conjugate (horseradish peroxidase-conjugated detection antibody) was then added to each well, and the plate was incubated again at 37°C for the period specified in the kit insert. A second wash cycle was performed, after which 3,3′,5,5′-tetramethylbenzidine (TMB) substrate solution was added to each well, and the plate was incubated at room temperature in the dark for the time specified by the manufacturer. Stop solution was then added to terminate the enzymatic reaction. Optical density (OD) was measured at 450 nm using a microplate spectrophotometer within 15 min of adding the stop solution.

#### Calculation of cutoff value and interpretation of results

For each plate run, the cutoff OD value was calculated according to the formula specified in the kit insert, derived from the mean OD readings of the negative controls included in that run.

The run criteria were as follows:

1. The individual absorbance value of negative controls should be less than 0.1.
2. The individual absorbance value of positive controls should be more than 1.0.

The cutoff value was calculated by adding 0.2 to the average absorbance value of the negative control.

A specimen was classified as positive for dengue NS1 antigen if its OD value was equal to or greater than the calculated cutoff value, and as negative if its OD value was below the cutoff. All plate runs were validated by confirming that the positive and negative control OD readings fell within the ranges specified by the manufacturer. Any run in which controls fell outside acceptable ranges was repeated.

## RESULTS

A total of 3,488 patient samples were included in the analysis, collected between 2 January 2023 and 31 December 2024. Of these, 1,036 samples (29.71%) tested positive for dengue by NS1Ag-ELISA and were considered confirmed cases, while 2,452 samples (70.29%) were ELISA-negative. Comparison of RDT against ELISA revealed 988 true-positive results and 2,274 true-negative results. There were 178 false-positive cases in which RDT yielded a positive result despite a negative ELISA, and 48 false-negative cases in which RDT failed to detect dengue in ELISA-positive samples.

The diagnostic performance of NS1Ag - RDT relative to NS1Ag-ELISA is summarized in Tables 1 and 2.

**TABLE 1:** Distribution of dengue patient samples tested using NS1 antigen-based ELISA^*a*^^,^^*b*^

Total samples	ELISA positive	ELISA negative
3,488	1,036	2,452

^*a*^ Of 3,488 samples analyzed, 1,036 were ELISA positive, and 2,452 were ELISA negative.

^*b*^ ELISA, NS1Ag-based enzyme linked immunosorbent assay.

**TABLE 2:** Cross-tabulation of NS1 antigen-based rapid diagnostic test (RDT) results against NS1Ag-based ELISA as the reference standard^*a*^

	ELISA		
		(−)	(+)
RAPID (RDT NS1Ag)	(−)	2,274	48
	(+)	178	988

^*a*^ Based on overall data, the NS1Ag-RDT demonstrated a sensitivity of 95.36% (95% CI: 93.91–96.49%) and a specificity of 92.74% (95% CI: 91.65–93.70%) relative to NS1Ag-ELISA. The positive predictive value (PPV) was 84.73% (95% CI: 82.56–86.68%) and the negative predictive value (NPV) was 97.93% (95% CI: 97.27–98.44%). The positive likelihood ratio (LR+) was 13.14 and the negative likelihood ratio (LR−) was 0.05, indicating strong diagnostic utility of the RDT for both ruling in and ruling out dengue infection when used alongside ELISA.

The 3,488 samples were distributed across four seasons as follows: summer (March–May 2023 and 2024) comprised 800 samples; monsoon (June–September 2023 and 2024) comprised 1,276 samples; autumn or post-monsoon (October–November 2023 and 2024) comprised 630 samples; and winter (January, February, and December 2023 and 2024) comprised 782 samples.

Season-wise 2 × 2 contingency tables of NS1Ag-RDT results against NS1Ag-ELISA as the reference standard are given below:

Furthermore, the sensitivity and specificity of RDT compared to ELISA on a seasonal basis revealed the following outcomes, as is depicted in the chart given below (Fig. 1).

**Fig 1:**
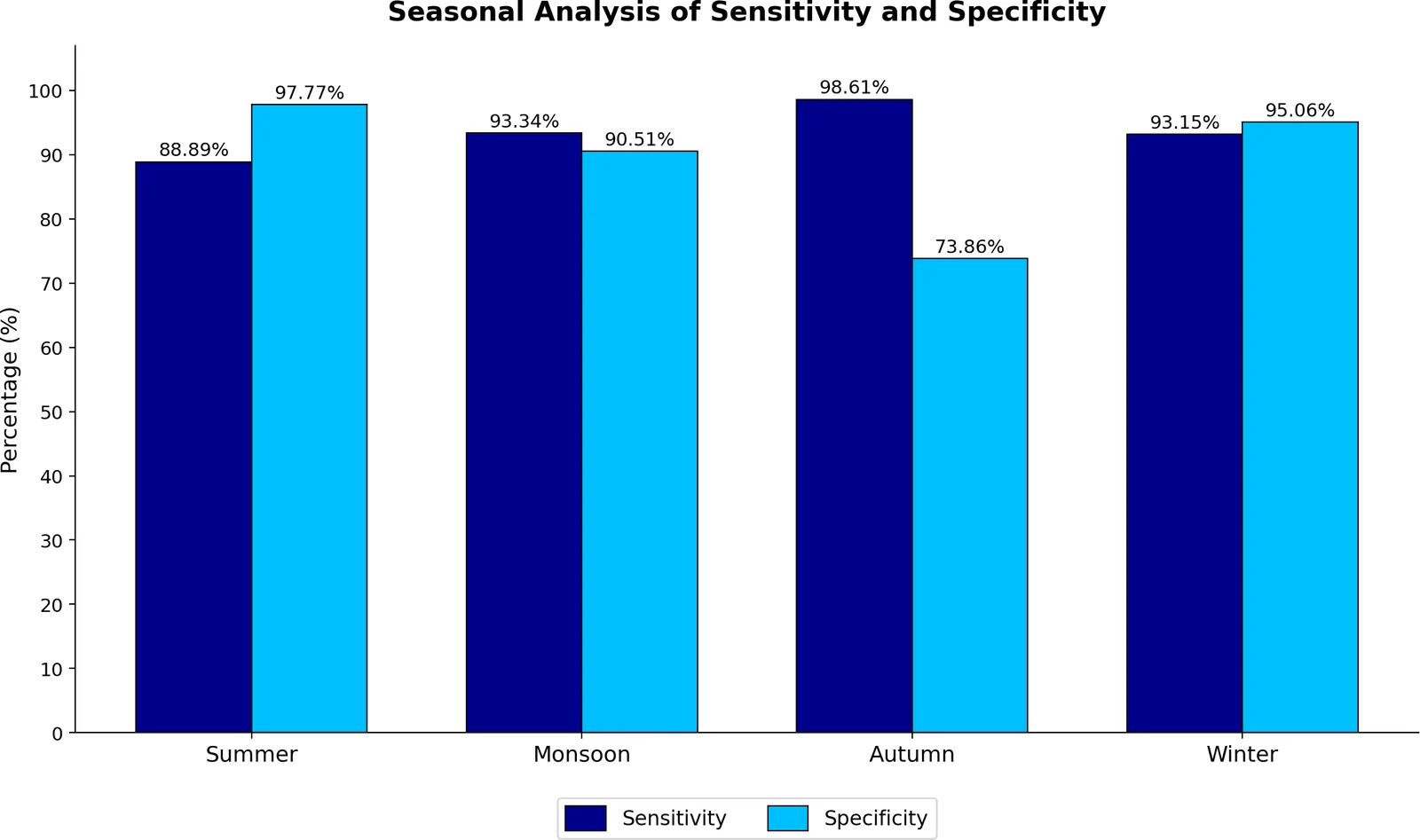
Illustration of the month-wise comparison of sensitivity and specificity of the NS1 antigen-based rapid diagnostic test (RDT) in comparison with NS1Ag-based ELISA across four different seasons. Sensitivity and specificity values are expressed as percentages, highlighting temporal variation in NS1Ag-based RDT performance relative to the reference standard, NS1Ag-based ELISA.

The seasonal analysis demonstrated notable variation in the diagnostic performance of NS1 antigen-based RDTs when compared with ELISA. During the summer months (March–May), the RDT showed a sensitivity of 88.89% and a specificity of 97.77%. Performance improved in the monsoon season (June–September), where sensitivity increased to 93.34%, although specificity decreased to 90.51%. The highest sensitivity was recorded in the autumn/post-monsoon period (October–November), reaching 98.61%; however, this coincided with the lowest specificity of 73.86%. In the winter months (January-February and December), diagnostic performance stabilized with sensitivity at 93.15% and specificity at 95.06%. These findings suggest that RDT accuracy fluctuates seasonally, with particularly strong sensitivity but weaker specificity during peak transmission periods.

Chi-square analysis demonstrated that seasonal differences in both sensitivity and specificity were statistically significant. Sensitivity varied significantly across seasons (chi-square = 19.06, df = 3, *P* = 0.0003), as did specificity (chi-square = 145.46, df = 3, *P* < 0.0001), indicating that seasonal factors meaningfully influenced the diagnostic performance of NS1Ag-RDT relative to NS1Ag-ELISA.

A post hoc power analysis confirmed that the study was adequately powered to detect clinically meaningful differences in diagnostic performance. With 1,036 ELISA-positive samples, the study achieved 100% power to detect a difference in sensitivity from a null value of 0.85 at a significance level of 0.05. With 2,452 ELISA-negative samples, the study similarly achieved 100% power for specificity estimation, confirming the adequacy of the sample size for the primary objectives.

Among the 226 total discordant results (178 false positives [FPs] and 48 false negatives [FNs]), a clear seasonal pattern was observed. The proportion of discordant results relative to total seasonal samples was lowest in summer at 2.6% (21 out of 800 samples; FP = 17, FN = 4) (Table 3), increased in winter at 5.1% (40 out of 782 samples; FP = 35, FN = 5) (Table 4), and was highest in monsoon and autumn at 8.4% (107 out of 1,276 samples; FP = 74, FN = 33) and 9.2% (58 out of 630 samples; FP = 52, FN = 6) (Tables 5 and 6), respectively. The elevated false positive rate in autumn, accounting for 52 out of 199 RDT-positive results in that season (26.1%), corresponds directly with the lowest observed specificity of 73.87% during that period. The clustering of false positives in the post-monsoon months is consistent with the known persistence of NS1 antigen cross-reactivity and non-specific reactions in high-transmission periods. As this was a register-based retrospective study, patient-level variables including age group, clinical severity, and symptom onset timing were not captured in the laboratory register and therefore could not be analyzed in relation to discordant results. This is acknowledged as a limitation of our study.

**TABLE 3:** Summer (March–May; *n* = 800)^*a*^

	ELISA		
		(−)	(+)
RAPID (RDT NS1Ag)	(−)	747	4
	(+)	17	32

^*a*^ Analysis of the Summer interval (*n* = 800) indicated that the NS1Ag-RDT achieved a sensitivity of 88.89% (95% CI: 74.68–95.59%) and a specificity of 97.77% (95% CI: 96.47–98.61%). The positive predictive value (PPV) was determined to be 65.31% (95% CI: 51.31–77.08%), with a negative predictive value (NPV) of 99.47% (95% CI: 98.64–99.79%). Additionally, the positive likelihood ratio (LR+) was 39.95 and the negative likelihood ratio (LR−) was 0.11.

**TABLE 4:** Winter (January, February, December; *n* = 782)^*a*^^,^^*b*^

	ELISA		
		(−)	(+)
RAPID (RDT NS1Ag)	(−)	674	5
	(+)	35	68

^*a*^ Evaluation of the Winter season (*n* = 782) revealed that the NS1Ag-RDT yielded a sensitivity of 93.15% (95% CI: 84.95–97.04%) and a specificity of 95.06% (95% CI: 93.21–96.43%). The positive predictive value (PPV) was 66.02% (95% CI: 56.44–74.44%), while the negative predictive value (NPV) was 99.26% (95% CI: 98.29–99.69%). A calculated positive likelihood ratio (LR+) of 18.87 and a negative likelihood ratio (LR−) of 0.07 further characterize the diagnostic performance during this period.

^*b*^ ELISA, NS1Ag-based enzyme linked immunosorbent assay; RDT NS1Ag, NS1Ag-based rapid diagnostic test.

**TABLE 5:** Monsoon (June–September; *n* = 1,276)^*a*^

	ELISA		
		(−)	(+)
RAPID (RDT NS1Ag)	(−)	706	33
	(+)	74	463

^*a*^ In the Monsoon season (*n* = 1,276), the NS1Ag-RDT yielded a sensitivity of 93.34% (95% CI: 90.80–95.22%) and a specificity of 90.51% (95% CI: 88.25–92.38%). The positive predictive value (PPV) reached 86.22% (95% CI: 83.05–88.88%), while the negative predictive value (NPV) was 95.53% (95% CI: 93.80–96.80%). A calculated positive likelihood ratio (LR+) of 9.84 and a negative likelihood ratio (LR−) of 0.07 further define the diagnostic utility during this peak interval.

**TABLE 6:** Autumn or post-monsoon (October–November; *n* = 630)^*a*^

	ELISA		
		(−)	(+)
RAPID (RDT NS1Ag)	(−)	147	6
	(+)	52	425

^*a*^ In the Autumn or post-monsoon period (*n* = 630), the NS1Ag-RDT exhibited a sensitivity of 98.61% (95% CI: 97.00−99.36%) and a specificity of 73.86% (95% CI: 67.35−79.48%). During this interval, the positive predictive value (PPV) reached 89.10% (95% CI: 85.98−91.59%), while the negative predictive value (NPV) was 96.08% (95% CI: 91.71−98.19%). The calculated positive likelihood ratio (LR+) was 3.77, and the negative likelihood ratio (LR−) was 0.02.

## DISCUSSION

Dengue is a mosquito-borne viral hemorrhagic fever with a rising global burden and remains particularly endemic in India, Southeast Asia, and Africa (2). India continues to experience a year-on-year increase in reported dengue incidence and dengue-related deaths (3, 9–12). This global increase highlights the need for rapid diagnostic tools, wider screening coverage, and stronger evidence supporting current diagnostic algorithms.

At present, dengue diagnosis in most laboratories involves viral isolation, nucleic acid amplification assays for detecting the viral genome (such as RT-PCR, NASBA, or real-time RT-PCR), and detection of dengue virus-specific antibodies. After the onset of illness, the virus is detectable in serum, plasma, circulating blood cells, and immune tissues for approximately 2–7 days, corresponding to the febrile phase. Molecular assays like RT-PCR have largely replaced virus isolation as the standard method for detecting dengue virus in acute-phase samples (13). Several studies have evaluated the diagnostic efficacy of NS1 antigen-based rapid diagnostic tests (RDTs) and enzyme-linked immunosorbent assay (ELISA) compared with the gold standard RT-PCR (1, 14–18). However, few have directly compared RDT and ELISA performance, and even fewer studies have focused on the Indian setting.

Rapid diagnostic tests (RDTs) are widely used in India for dengue diagnosis due to limited ELISA availability (1). Multiple studies consistently report higher accuracy for NS1Ag detection than IgM. Garg et al. showed IgM sensitivity of 27.8–77.7% and specificity of 50–100% whereas NS1 demonstrated 99% sensitivity and 96% specificity (1). Similar high NS1 performance was noted by Reddy et al. with sensitivities of 90.11% compared to 98.45% for ELISA (19) and by Kulkarni et al. who found high NS1 sensitivity and specificity despite lower combined IgM plus NS1 sensitivity of 55.7–76.6% (5), while Shukla et al. observed 99.2% sensitivity and 96.0% specificity, with more than 90% serotype-specific sensitivity and specificity across DENV-1, -2, and -3 (6). Blacksell et al. reported NS1 RDT specificity typically above 90–100% and sensitivity ranging from 48% to 92% in primary and from 70% to 93% in secondary dengue (17), consistent with Liu et al. who showed better performance in primary infection (20). Naidu et al. reported NS1 RDT sensitivity and specificity of 87.50% and 98.52% vs RT-PCR and 93.75% and 100% for NS1 ELISA (21), and Mahajan et al. supported better NS1 performance than IgM with improved accuracy when combined in a single kit (22). Another study showed higher NS1 positivity than qRT-PCR in primary infection 97.8% vs 84.8% and 94.8% vs 71.8% but qRT-PCR superiority in secondary infection 58.1% vs 48.4% and 50.0% vs 41.4% (23). These studies show that NS1 RDTs provide rapid and fairly accurate diagnosis early in illness, particularly for primary infections (1, 5, 6, 17, 19–27).

The NVBDCP endorses NS1 antigen-based RDTs for early diagnosis, followed by ELISA for confirmation in suspected dengue cases, especially in outbreak-prone settings. Our study found that among 3,488 samples, 1,036 were ELISA-positive and 2,452 were ELISA-negative. The corresponding RDT results yielded 988 true positives, 178 false positives, 2,274 true negatives, and 48 false negatives. These results demonstrate that NS1Ag-RDTs achieved a sensitivity of 95.36% and specificity of 92.74% relative to ELISA, consistent with findings reported in the literature, thereby providing evidence to support these national guidelines by demonstrating the high diagnostic accuracy of NS1Ag-RDTs.

The seasonal trend analysis in our study revealed important diagnostic fluctuations. Sensitivity of RDTs was highest in autumn/post-monsoon (98.61%), a known peak transmission season, while specificity dropped to its lowest (73.86%) during the same period. In contrast, during summer, RDTs had the lowest sensitivity (88.89%) but the highest specificity (97.77%). These findings indicate that diagnostic reliance on RDTs may need stratification by season, with supplementary IgM ELISA or RT-PCR recommended during high-transmission periods. Such seasonal variability aligns with documented differences in serotype circulation, secondary infections, and viral load changes across the year (18, 25, 28–33).

NS1-based tests, including both RDTs and ELISA, demonstrate the highest diagnostic performance within the first 5–7 days of illness, while IgM-based ELISA becomes more reliable beyond the acute febrile phase. The findings of our study align with this established temporal pattern and support the use of NS1 testing early in the course of infection. However, multiple studies have reported a notable reduction in the diagnostic accuracy of NS1-based assays (both RDTs and ELISA), and antidengue IgM detection by RDTs in late acute stages and in secondary dengue infection (1, 5, 22). Therefore, combining NS1 antigen tests with antidengue IgM-based RDTs and ELISA enhances diagnostic yield across different phases and forms of dengue infection, ensuring more comprehensive case detection.

This study has certain limitations. First, while viral isolation and RT-PCR remain established gold standards for definitive confirmation due to their ability to detect viral nucleic acids directly, our study utilized NS1Ag-based ELISA as the reference standard. This approach is grounded in practical considerations common in Indian tertiary care settings; molecular assays like RT-PCR often require specialized infrastructure and trained personnel that are not consistently accessible for routine diagnostics. In alignment with NVBDCP guidelines, which endorse NS1Ag-ELISA for confirmation in resource-constrained environments, several studies in the Indian context have similarly adopted this reference framework (5, 19, 21, 34). Readers should, however, interpret the reported diagnostic accuracy with caution. The absence of molecular validation means a small fraction of ELISA-positive samples may not reflect true virological infection, potentially influencing sensitivity and specificity estimates. Future research incorporating RT-PCR alongside ELISA would strengthen the evaluation of NS1Ag-RDT performance (1, 5, 16).

Second, while our study evaluates seasonal diagnostic variability, it does not analyze seasonal changes in dengue incidence or serotype prevalence, which may further influence test performance.

Although molecular characterization of DENV serotypes was not performed as part of the routine laboratory protocol at this institution due to infrastructure constraints, existing surveillance data at the regional and national levels provide a critical framework for interpreting our findings. These published reports allow for a contextual understanding of the likely circulating serotypes during the 2023–2024 study period and their potential influence on the seasonal fluctuations observed in NS1Ag-RDT diagnostic performance.

The epidemiological landscape of dengue serotypes in Mumbai and broader Western India is characterized by significant temporal dynamics. Previous investigations have documented the co-circulation of all four DENV serotypes (DENV-1 to DENV-4) within the city, with annual shifts in the dominant serotype frequently observed (35). In Maharashtra, reports from 2017 and 2018 highlighted the detection of all four serotypes within single transmission cycles, with DENV-1 and DENV-3 alternating as the primary strains, reflecting a rapid turnover even within localized geographic areas (36). On a national scale, DENV-2 has increasingly displaced DENV-1 as the most prevalent serotype since 2019. Notably, a novel DENV-2 Genotype IV Lineage III strain was identified across central and western regions through 2023, coinciding with the initial year of our retrospective analysis (10, 37–39). Urban centers in Western India are recognized as hyperendemic zones with high serotype diversity driven by fluctuations in vector density and population immunity (1); surveillance data spanning 2014 to 2022 confirm a DENV-2 predominance of 66.40%, followed by DENV-3 at 19.82% (40).

Such serotype-specific dynamics appear to have a direct bearing on the seasonal variability in RDT accuracy identified in our results. NS1 antigen kinetics, specifically the intensity and duration of antigenemia, vary significantly across serotypes. DENV-2 infections, in particular, are associated with higher peak NS1 concentrations and a more persistent antigenaemic phase, which may enhance RDT sensitivity while simultaneously increasing the risk of non-specific cross-reactivity during periods of high viral burden. This provides a plausible biological basis for the patterns observed in our study: in the autumn or post-monsoon months, the peak transmission interval in Mumbai was likely dominated by DENV-2, where sensitivity reached a maximum of 98.61% while specificity dropped to a study-low of 73.86% (39, 41, 42). The 26.1% false-positive rate observed during this peak season is consistent with the anticipated high NS1 levels and potential cross-reactive signals during intense transmission periods. In contrast, during the summer, when transmission and DENV-2 prevalence are minimal, specificity reached 97.77% while sensitivity was at its lowest (88.89%), reflecting reduced NS1 concentrations and a narrower diagnostic window (35, 36, 43). The monsoon and winter periods exhibited intermediate diagnostic outcomes, likely reflecting transitional transmission intensities and serotype shifts (40, 42).

While these observations are inferential given the absence of direct serotyping data, they align with established literature on serotype-dependent diagnostic kinetics and support the hypothesis that seasonal DENV shifts contribute to the documented performance variability. Future prospective research incorporating RT-PCR-based serotyping at this facility would be vital to definitively validate these relationships.

The results obtained from this analysis offer meaningful insights for clinical diagnostic applications within endemic and resource-constrained environments. Given the robust overall sensitivity of 95.36% observed for NS1Ag-RDT, its utilization as a primary screening instrument remains justified, especially in peripheral healthcare facilities where ELISA capabilities are restricted or where rapid turnaround is vital for immediate clinical management. Furthermore, the substantial negative predictive value (97.93%) indicates that negative results may effectively serve to rule out acute dengue during periods of lower transmission, thereby optimizing resource distribution and minimizing unnecessary clinical referrals.

Third, this is a single-center study, which may not fully capture serotype distribution or epidemiological variability across different Indian regions. Conducting multicentric studies across diverse ecological zones would strengthen the generalizability of these findings.

Nevertheless, the identified seasonal fluctuations in diagnostic accuracy necessitate a more nuanced interpretation of RDT outcomes during specific intervals. In the autumn or post-monsoon phase, RDT specificity declined to 73.86%, suggesting that approximately 26% of positive results were discordant with ELISA findings. Within the framework of resource-limited public hospitals, where clinical pathways often rely on standalone RDT data, such elevated false-positive rates during peak transmission may lead to overdiagnosis and inappropriate patient management. Consequently, a seasonally stratified diagnostic approach is recommended: in summer and winter, when specificity remains above 95%, RDT-positive results may be deemed reliable for initial triage, with ELISA reserved for complicated cases. Conversely, during the monsoon and autumn months, characterized by higher transmission and lower specificity, ELISA confirmation for all RDT-positive samples is encouraged. In settings lacking ELISA infrastructure, the marked difference in positive likelihood ratios, ranging from 39.95 in summer to 3.77 in autumn, underscores that post-monsoon positive results carry a lower probability of true infection. These temporal variations should be integrated into institutional protocols and reflected in laboratory reporting to ensure precise diagnostic decision-making.

Overall, the present study reinforces the diagnostic accuracy and clinical practicality of NS1Ag-RDTs as rapid, accessible, and reliable tools for early dengue detection in endemic, resource-constrained healthcare settings. At the same time, it highlights the need to integrate IgM-based diagnostic tests (RDTs and ELISA) to ensure accurate detection of late acute dengue infection and secondary infections. The observed seasonal variation also warrants the need for region- and season-specific diagnostic strategies and further multicentric research incorporating RT-PCR for serotype correlation and validation.

### Conclusion

Dengue continues to contribute significantly to global and national morbidity and mortality. The findings of this study substantiate the diagnostic utility of rapid diagnostic NS1Ag testing, particularly in time- and resource-limited settings, demonstrating high accuracy when compared with the NS1Ag-based enzyme-linked immunosorbent assay (ELISA). This evidence reinforces the validity of existing national guidelines for the diagnosis and management of dengue in India, while also supporting the need to incorporate antidengue IgM-antibody-based tests and RT-PCR for detecting secondary and late acute dengue infections.

Further multicentric and regionally diverse studies incorporating RT-PCR as the gold standard are warranted to comprehensively evaluate and optimize the diagnostic performance of RDT and ELISA, thereby strengthening evidence-based dengue surveillance and control efforts.
